# Editorial: Outcomes in subarachnoid hemorrhage

**DOI:** 10.3389/fneur.2023.1186473

**Published:** 2023-05-03

**Authors:** Sarah E. Nelson, Isabel Fragata, Matthew Rowland, Airton Leonardo de Oliveira Manoel

**Affiliations:** ^1^Department of Neurosurgery, Mount Sinai Health System, New York, NY, United States; ^2^Department of Neurology, Mount Sinai Health System, New York, NY, United States; ^3^Centro Hospitalar Universitário de Lisboa Central, Lisbon, Portugal; ^4^Nova Medical School, Lisbon, Portugal; ^5^Wellcome-Wolfson Institute for Experimental Medicine, Department of Medicine, Dentistry and Biomedical Sciences, Queen's University, Belfast, United Kingdom; ^6^Novartis Pharmaceuticals, London, United Kingdom; ^7^Sultan Qaboos Comprehensive Cancer Care and Research Centre, Department of Intensive Care Medicine, Muscat, Oman

**Keywords:** subarachnoid hemorrhage, outcomes, research, prognostication, biomarker

Welcome to our ResearchTopic on “*Outcomes in subarachnoid hemorrhage*” in *Frontiers in Neurology*. Although aneurysmal subarachnoid hemorrhage (SAH) represents only 5% of all types of stroke, it carries the highest mortality and disability rates (1–3). Moreover, because SAH affects predominantly young adults, it is associated with huge economic and social burden (4). In addition, a considerable number of patients with an apparent full recovery may experience residual neurocognitive impairment, which are subtle and difficult to recognize, however it may prevent them from returning to work and having a normal life (5). Therefore, the current Research Topic may be of interest to those involved in treating or conducting research in SAH.

Some of the enclosed manuscripts discuss the relationship between clinical factors or biomarkers and SAH outcome directly (Zhang et al.; Wen et al.; Wang et al.). For instance, Zhang et al. found that the C-reactive protein to lymphocytes ratio was associated with unfavorable functional outcome in aneurysmal SAH. Wen et al. observed that parenchymal blood volume maps as acquired by C-arm flat-panel detector CT appear to be associated with discharge outcomes. Wang et al. evaluated factors associated with occurrence of delayed cerebral ischemia and prognosis of patients with aneurysmal SAH associated with hydrocephalus, noting that high Hunt and Hess Grade and surgical clipping were independent risk factors for delayed cerebral ischemia, while older age, higher Hunt and Hess Grade, higher C reactive protein, and increased neutrophil levels were independently associated with unfavorable outcomes at 6 months. Another interesting article discusses the role of the double microcatheter technique as a possible safe and effective treatment for anterior circulation ruptured aneurysms (Zhao et al.). These articles thus add to the literature regarding SAH treatment and prognostication, the latter of which is still fraught with uncertainty (6).

Additionally, two articles included in this series explore the relevance of clinical factors in relation to SAH patients' clinical course (Yuan et al.; Santana et al.), including the development of pneumonia and contribution to early brain injury. For instance, Yuan et al. noted that post-operative pneumonia was more commonly found in those who had undergone surgical clipping vs. endovascular coiling for aneurysm securement and furthermore noted several risk factors for post-operative pneumonia in each of the surgical clipping and endovascular coiling groups. Also, Santana et al. reviewed the literature on glycemic management and SAH, showing an association between dysglycemia and outcome, and the fact that better knowledge regarding glucose patterns and management surrounding aneurysmal SAH is needed. These articles thus contribute knowledge surrounding the systemic complications that commonly occur in SAH patients, suggesting possible ways in which these medical conditions could be addressed.

Since outcomes can vary even within a single country, Shah et al. presented data examining clinical factors in different areas of the US in relationship to SAH outcomes, and described the variability in SAH care across the country. They also found several clinical factors that appear to affect SAH outcome (Shah et al.). Finally, Andersen, Presseau et al. review what, when, and how to measure in choosing relevant outcomes for aneurysmal SAH as well as initiatives to improve the selection of these outcomes. In a later article, Andersen, English et al. present the results of a survey to assess these outcomes' priorities for different SAH stakeholders (including patients and clinicians). They found that there were several viewpoints concerning optimal aneurysmal SAH outcome measures though patient-reported quality of life was deemed the most important outcome measure (Andersen, English et al.). These articles are important because they point out not only that clinical care in SAH management still appears to be variable (even within one country), but also that choosing appropriate outcomes for this condition is critically important, warranting a thorough evaluation. Hopefully, the articles included in this *Frontiers in Neurology* Research Topic prove to be useful, and in some cases potentially provide the impetus for further research into the topic of SAH outcomes.

## Author contributions

SN drafted and edited the manuscript. IF, MR, and AO critically reviewed the manuscript. All authors contributed to the article and approved the submitted version.

## References

[1] Nieuwkamp DJ Setz LE Algra A Linn FHH de Rooij NK Rinkel GJE. Changes in case fatality of aneurysmal subarachnoid haemorrhage over time, according to age, sex, and region: A meta-analysis. Lancet Neurol. (2009) 8:635–42. 10.1016/S1474-4422(09)70126-7 19501022

[2] Manoel ALd Mansur A Silva GS Germans MR Jaja BNR Kouzmina E. Functional outcome after poor-grade subarachnoid hemorrhage: A single-center study and systematic literature review. Neurocrit Care. (2016) 25:338–50. 10.1007/s12028-016-0305-3 27651379

[3] Manoel ALd Goffi A Marotta TR Schweizer TA Abrahamson S Macdonald RL. The critical care management of poor-grade subarachnoid haemorrhage. Crit Care. (2016) 20:21. 10.1186/s13054-016-1193-9 26801901 PMC4724088

[4] Thompson JC Chalet F-X Manalastas EJ Hawkins N Sarri G Talbot DA. Economic and humanistic burden of cerebral vasospasm and its related complications after aneurysmal subarachnoid hemorrhage: A systematic literature review. Neurol Ther. (2022) 11:597–620. 10.1007/s40120-022-00348-6 35441974 PMC9095797

[5] Al-Khindi T Macdonald RL Schweizer TA. Cognitive and functional outcome after aneurysmal subarachnoid hemorrhage. Stroke. (2010) 41:e519–36. 10.1161/STROKEAHA.110.581975 20595669

[6] Connolly ESC Jr Rabinstein AA Carhuapoma JR Derdeyn CP Dion J Higashida RT. Guidelines for the management of aneurysmal subarachnoid hemorrhage: A guideline for healthcare professionals from the American Heart Association/American Stroke Association. Stroke. (2012) 43:1711–37. 10.1161/STR.0b013e3182587839 22556195

